# Association of nutritional status with institution type, residence area and oral health among school children in India

**DOI:** 10.6026/973206300220216

**Published:** 2026-01-31

**Authors:** Harshitha Yaramala, Haritha K, Punithavathy R, Satyam M, Sri Ramya M, Prasanthi BB

**Affiliations:** 1 Department of Pediatric and Preventive Dentistry, Lenora Institute of Dental Sciences, Rajahmundry, Andhra Pradesh, India

**Keywords:** Gingival inflammation, plaque status, nutritional status, school children, rural area

## Abstract

Poor nutritional status among rural adolescents contributes significantly to increased plaque accumulation and gingival inflammation. Therefore, it is of interest to compare and assess the nutritional status based on their place of residence and the institution type and its potential consequences on the plaque and gingival condition among high school children. Two hundred 12 -15 years old school children of 100 each of the government and the private schools of the rural area known as Rajanagaram were chosen to take part in the study. A Nutrition Evaluation Chart and Plaque and Gingival Index were used to check the selected children on their nutritional status and plaque and gingival status, respectively. The two groups differed greatly in terms of the plaque and nutritional status. Adolescents from lower socioeconomic, government school backgrounds in a rural area show significantly poorer nutrient scores and higher plaque scores than their private school counterparts.

## Background:

Proper balance of diet offers optimum health to an individual [1]. Poor diet is the main etiological factor of all the diseases of deficiency. In most cases, children are the most impacted. It is calculated that 85 percent of children who have 4070 percent of preschool children in India, are having dental caries [2]. It is only under health promotion programs that the health of people can be improved the best way that can be promoted are through schools. Most of the nations have established screening of early detection and promotion into the education system [3]. The dentists have one-on-one personal instruction method that is common in disseminating oral hygiene messages [4]. Therefore, it is of interest to evaluate nutritional status among institution type and rural residence. Further, its association with plaque and gingival status among 12-15 year old schoolchildren in India is also explored.

## Materials and Methods:

The current research was done in the government and the private school premises of rural areas in the East Godavari district. The Institutional Ethical Committee provided ethical clearance to the commencement of the study. Inclusion Criteria were healthy children between the age group of 1215 years. Those students who are available during screening. Children are willing to cooperate and ready to do it. Exclusion Criteria were; Children who had any physical or mental disabilities and parents who did not consent to participate. To identify a discrepancy in the groups, a sample size of 200 was decided with an 80 percent power of the study at the significant level of 0.05. The study was well informed of the procedure and informed consent was taken prior to the study. The samples in government and private schools of Rajanagaram were selected through simple random sampling method and checked on their nutritional condition and plaque and the gingival index of the samples in both groups GROUP 1: Government school children (n = 100) and GROUP 2: Private school children (n = 100). The plaque scores were calculated by the use of the Plaque Index given by Sillness and Loe (1964). The teeth and gingiva were isolated with cotton rolls and a mouth mirror, source of light and dental explorer were used to examine six index teeth. The Gingival Index of Loe and Sillness (1963) was the one according to which the gingival scores were calculated [5, 6]. In the case of the Nutritional Status Assessment, weekdays were considered so that the students were expected to record a 5-day diet journal. Once the diet diary of the two groups was collected, the nutrient scores (NS) of the two groups were computed with the help of the Nutrient Evaluation Chart. All the data collected were taken to analyze them statistically and the findings tabulated.

## Results:

The obtained data in the study were statistically analyzed using the Statistical Package for Social Sciences (SPSS Version 26.0). The results were expressed as mean values along with their standard deviation. The statistical tests used for intergroup comparison and intragroup comparison were done using independent t-tests & paired t-tests respectively, and a p-value < 0.05 was considered statistically significant, and a p-value < 0.01 was considered highly statistically significant. Table 1 shows a comparison of plaque, gingival and nutrient scores between government and private schools. There was a highly significant difference in plaque and nutrient scores at a p-value <0.01. Whereas there was no significant difference in gingival scores at a p-value <0.05.

## Discussion:

Dental cavities and periodontal disorders are very socially, economically, and financially challenging across the world. Despite the generous variety of preventive solutions, the main cause of these issues the dental plaque is a persistent problem. According to recent studies, other factors such as the rate of food intake by an individual could also contribute to the cariogenic nature of the oral environment. Thus, plaque and gingiva states may help to understand the cariogenicity of food and its effect on oral health [7]. The age group of 12 15 years old was selected in the study in line with the WHO criteria since 12 years old is considered as an age at which medical trends including dental caries can be internationally compared and tracked globally [8]. The results of the current study proved that there was a very strong difference in plaque score of the government school subjects compared to the plaque scores of the children at the private schools located in the rural areas with higher plaque scores of the children of the government school subjects. This can be compared to the Gaur *et al.* study that found out that the scores of gingival and plaque were higher in rural private schools [9]. A study carried out by Lindhe, Koch. also revealed the same outcome as the current study in which plaque and gingival scores decreased among children in the control group which was explained by the Hawthrone effect (non-specific treatment effect where shift in behavior is caused by motivation provided by the attention or care or interest acquired by observation and evaluation) [10]. The current research demonstrates that the difference between the nutritional scores in the existing study is quite high, and the nutritional score is higher in the case of the private schools [11,12-13]. Consequently, most students studying in government or state schools belong to less privileged socioeconomic backgrounds since their parents cannot afford the expenses of studying in the private school [4]. Differences that exist between the findings in this study could be attributed to the societal background of the researchers, sample of study, and or geographical location, which was regarded as an independent determinant and which consequently could offer some support to ethnic and potentially genetic differences [14, 15]. Literature has little research on the association between plaque and gingivitis health and nutritional status across geographic location and type of institution. Given the fact that improving oral health of children and raising awareness with regard to effective oral hygiene practices among all socioeconomic groups may help achieve better oral health outcomes, it is advisable to conduct additional research on the matter.

## Conclusion:

The participants showed get nutritional counseling and rudimentary oral health survey. Dietary counseling should take place at the school level as part of all the preventive oral care programs (team of health supervisors). More data is needed in a large scale that would help in the implementation and maintenance of health programs in schools.

## Figures and Tables

**Table 1:** Comparison of plaque, gingival and nutrient scores between government and private schools in rural area

**VARIABLE**	**GROUP**	**MEAN ± SD**	**P-VALUE**
Plaque scores	Government	0.260±0.197	0.007**
	Private	0.185±0.153	
Gingival scores	Government	0.132 ±0.144	0.078
	Private	0.122± 0.125	
Nutrient scores	Government	55.290±2.454	0.000**
	Private	68.350± 6.509	

## References

[1] Koziol-Kozakowska  A (2023). Children (Basel)..

[2] Amudha S (2021). Int J Clin Pediatr Dent..

[3] Pandey P (2021). J Int Soc Prev Community Dent..

[4] Barbosa MC (2021). Global pediatric health..

[5] Loe H, Silness J (1963). Acta Odontol Scand..

[6] Silness J, Loe H (1964). Acta Odontol Scand..

[7] Sosiawan A (2020). IJPHRD..

[8] Andegiorgish AK (2017). BMC Oral Health..

[9] Gaur AG (2023). Int J Clin Pediatr Dent..

[10] Vijayasekaram R (2020). J Global Oral Health..

[11] Suchitra MR (2021). Curr Res Nutr Food Sci..

[12] Kumar A, Khanna P. (2021). Int J Curr Microbiol App Sci..

[13] Sharma SJ (2018). J Comprehensive Health..

[14] Gaur A (2021). Int J Clin Pediatr Dent..

[15] Singh S, Talmale P (2023). J Oral Biol Cranio Res..

